# Small-incision phacotrabeculectomy versus phacoemulsification in refractory acute primary angle closure with cataract

**DOI:** 10.1186/s12886-015-0074-3

**Published:** 2015-07-29

**Authors:** Xu Hou, Dan Hu, Zhili Cui, Jian Zhou, Li Cai, Yusheng Wang

**Affiliations:** Eye Institute of Chinese PLA and Department of Ophthalmology, Xijing Hospital, Fourth Military Medical University, Xi’an, Shaanxi 710032 China

**Keywords:** Acute primary angle closure, Cataract, Phacotrabeculectomy

## Abstract

**Background:**

Acute primary angle closure (PAC) can be refractory to conventional treatment and intraocular pressure (IOP) is beyond control. Surgical intervention should be considered at the moment. The aim of the study was to compare small-incision phacotrabeculectomy (phacotrab, small-incision trabeculectomy combined with phacoemulsification) with phacoemulsification (phaco) in patients with refractory acute PAC and coexisting cataract.

**Methods:**

Analyzed 49 eyes (49 patients) with acute PAC and cataract received small-incision phacotrab (24 eyes) or phaco (25 eyes) randomly. All these cases were refractory to conventional treatment involved the use of preoperative topical IOP-lowering agents, corticosteroids, mannitol, methazolamide and paracentesis to reduce IOP. The effects on best corrected visual acuity (BCVA), IOP, anterior chamber depth (ACD), glaucoma medications, and complications were observed for twelve months.

**Results:**

After operation BCVA of 18 patients (75 %) in phacotrab group and 20 patients (80 %) in phaco group improved compared to preoperative vision. No statistically significant differences in mean BCVA were found between the two groups. The mean postoperative IOP levels at all follow up time points were lower than the mean preoperative IOP in each group (*P* <0.001). There was statistically significant difference in mean IOP between the two groups only at 12 months postoperatively (*P* = 0.006). The surgical success rate (without medications, IOP ≤ 21 mmHg) was 83.33 % (20 eyes) and 72 % (18 eyes) in phacotrab group and phaco group respectively at 12 months. No statistically significant differences in the mean ACD were found between the two groups. There were no serious intra- or post-operative complications in the two treatment groups.

**Conclusions:**

Besides phaco, small incision phacotrab may be another effective and safe choice in the treatment of patients with refractory acute PAC and coexisting cataract. Whether phacotrab is more effective in IOP control in the long term needs to be verified in the further.

## Background

Acute primary angle closure (PAC) is one of the most common ophthalmic emergencies. Many ocular risk factors have been shown to lead to acute PAC, including hyperopia, shallow anterior chamber depth (ACD), thick crystalline lens and short axial length [1]. Without treatment patients may develop primary angle closure glaucoma (PACG) and progressive vision loss [2]. Higher rates are reported in Asian populations compared to other races [3–6]. Prompt and effective intervention is required to control intraocular pressure (IOP), relieve pupil block, reopen the anterior angle, avoid further attacks and prevent damage to the optic nerve. The initial treatment of acute PAC include medications, paracentesis, laser peripheral iridotomy and etc. [7]. The role for surgical iridectomy and emergency trabeculectomy in the modern management of acute PAC is diminishing [8]. But in practice, some cases in Asian eyes are refractory to conventional treatment and IOP is beyond control. Surgical intervention may be required. In the study we defined “refractory” as conventional management did not achieve IOP control or relieve symptoms despite maximally tolerated medications or other non- surgically methods.

Primary lens extraction as treatment for acute PAC has been proved to relieve pupil block and result in good IOP control [9]. Because primary trabeculectomy early in acute PAC has a greater failure rate and higher incidence of complications, including shallow anterior chamber, malignant glaucoma, suprachoroidal hemorrhage, and endophthalmitis [10], trabeculectomy alone or combined lens extraction is occasionally used as the final step in the management of acute PAC, when all other modalities of treatment is failed [11]. While, with the development of new surgical technique and shift in views, it has been shown that trabeculectomy is safe and effective for PACG with persistent ocular hypertension [12]. The purpose of the current study was to compare primary small-incision phacotrabeculectomy (phacotrab) with phacoemulsification (phaco) on visual acuity (VA), IOP, ACD, glaucoma medications and complications in patients with refractory acute PAC and coexisting cataract.

## Methods

### Patients and inclusion criteria

This was a prospective analysis conducted at Xijing hospital in Xi’an, People’s Republic of China. The ethic and academic board of Xijing hospital approved the study and all procedures used conformed to the tenets of the Declaration of Helsinki. The informed consent documents were signed by all patients. 49 cases (49 eyes) with refractory acute PAC and coexisting cataract received small-incision phacotrab or phaco randomly as initial treatment from March 2011 to September 2013. The clinical diagnostic criteria for cataract were as follows: presence of nucleus sclerosis, cortical cataract, or subcapsular cataract which confirmed by slit-lamp examination and ultrasound biomicroscopy (UBM); visual acuity of 20/50 or worse. The diagnostic criteria for acute PAC were as follows: presence of typical symptoms including ocular pain, blurry or halo vision, nausea or vomiting; presenting IOP of more than 21 mmHg and the presence of typical signs including conjunctival injection, corneal epithelial edema, mid-dilated unreactive pupil, over 180 ° of iridotrabecular contact or peripheral anterior synechiae (PAS), and shallow anterior chamber. Patients with glaucomatous optic neuropathy, secondary ocular hypertention, surgery history, and any other ocular or system diseases were excluded. The serious opacity of refractive media, including corneal edema, inflammatory reaction in the anterior chamber and cataract could lead to the difficulties in fundus examination. As long as the refractive media was clear enough for fundus examination after surgery, we confirmed whether the patients met recruit criteria.

Sixteen patients (8 in each group) had one acute attack history and discontinuous IOP-lowing medical therapy previously. First line treatment such as systemic hyperosmotic agents (intravenous mannitol, 1 mg/kg), oral carbonic anhydrase inhibitors (methazolamide, 100 mg every eight hours), four kinds of topical IOP-lowering agents (Pilocarpine, timolol, brinzolamide and brimonidine) and other techniques like anterior chamber paracentesis were applied as long as the diagnosis of acute PAC was made. Laser iridotomy could not be performed because of corneal edema or mid-dilated unreactive pupil. Operation was done after 4 to 5 days of conventional management.

All patients presented significant cataract and PAS over 180 ° by indentation gonioscopy or UBM. They underwent a complete ocular examination before surgery, including best corrected visual acuity (BCVA), IOP, slit-lamp and fundus examination. IOL master or contact A-scan biomicroscopy was performed to measure the axial length and to calculate IOL power. The value of visual acuity was converted into that in the logarithm of the minimum angle of resolution (LogMAR).

### Surgery technique

All procedures were performed by one surgeon (D.H.). The surgical procedure in brief was as follows: after local anesthesia and sterile procedure, all patients underwent an anterior chamber paracentesis, 5 min later a standard phacoemulsification was performed through a 3-mm clear corneal incision. After a foldable hydrophobic acrylic IOL (XLSTABI ZO, Carl Zeiss) implantation and removal of viscoelastic material, the pupil was contacted by intracameral carbachol. The clear corneal incision was hydrosealed or sutured with one stitch of 10–0 Nylon, depending on the final wound condition. For phacotrab group, a fornix-based conjunctival flap (width: 5 mm) was used for trabeculectomy. A partial-thickness (approximately 50 % depth) triangular scleral flap was prepared, measuring approximately 3 mm at the limbus. Mitomycin C (0.4 mg/mL) was placed under the sclera flap for 2 to 3 min before irrigation with balanced salt solution. A sclerostomy was created using a Kelly Descemet’s punch, followed by a surgical peripheral iridectomy. The sclera flap was closed with 1 interrupted 10–0 nylon suture on the top and 2 releasable sutures on the waist. The fornix-based conjunctival flap was closed with continuous suture. Postoperative medications included tobramycin drops and prednisolone acetate ophthalmic suspension were administrated six times a day and reduced within 4 to 6 weeks depending on the degree of postoperative inflammation.

### Surgical outcomes

Postoperative data regarding BCVA and IOP (primary outcome measures), ACD, glaucoma medications and complications (secondary outcome measures) were obtained on week 1 and month 1, 3, 6, 9 and 12. UBM was performed at 6 month to evaluate ACD. Surgical success was defined as IOP ≤ 21 mmHg without glaucoma medications. Sutures removal and bleb needling to improve bleb function were not considered failure of the procedure.

### Statistical analysis

Statistical analysis was performed using SPSS software version 17 (SPSS Inc, Chicago, Illinois, USA). Continuous variables were expressed as mean ± standard deviation (SD), and categorical data were represented by number (n) and percentage (%). Parametric variables were analyzed using analysis of variance (ANOVA). Variables were compared using a paired *t* test. *P* value of <0.01 was considered statistically significant.

## Results

### Baseline characteristics

Of the 49 patients recruited in the study, 24 underwent phacotrab and 25 age- and sex-matched subjects underwent phaco. The patient demographics and ocular characteristics were showed in Table 1.

**Table 1:** Patient demographics and ocular characteristics of the two treatment groups

	Phacotrab group	Phaco group	*P* Value
No. of eyes	24	25	
Mean age ± SD (yrs)	62.38 ± 9.45 (45–78)	62.32 ± 8.48 (47–79)	0.98e*
Male: female ratio	5:19	5:20	0.94^†^
Axial length (mm)	21.54 ± 0.90 (20.5-23)	21.77 ± 1.06 (20.3-23.3)	0.41*
Lens thickness (mm)	4.64 ± 0.45 (3.83-5.36)	4.68 ± 0.48 (3.90-5.51)	0.78*

SD = standard deviation, *Student *t* test, ^†^Chi-square test

### Visual acuity

**Fig. 1:**
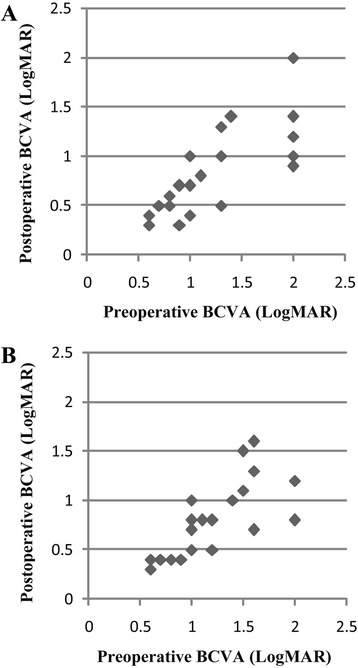
Comparison of patients’ pre- and post-operative best corrected visual acuity of the two treatment groups at six months. **a**: phacotrab group; **b**: phaco group

The pre- and post-operative BCVA (converted into LogMAR) of the two treatment groups were shown in Table 2. No statistically significant differences in mean BCVA were found between the two groups. 18 patients (75 %) in phacotrab group and 20 patients (80 %) in phaco group had improved VA on Snellen’s chart. In phacotrab group 4 patients retained the same initial VA, and 2 patients had light perception (LP) before and after surgery. In phaco group 3 patients retained the same initial VA, and 2 patients had LP before and after surgery. (Fig. 1, LP not shown)

**Table 2:** Pre- and Post-operative BCVA of the two treatment groups

LogMAR BCVA	Phacotrab group	Phaco group	*P* Value
Mean preoperative ± SD	1.21 ± 0.49	1.23 ± 0.43	0.93*
Mean postoperative ± SD (6 mos)	0.82 ± 0.44	0.80 ± 0.38	0.91*
Mean postoperative ± SD (12 mos)	0.82 ± 0.41	0.80 ± 0.39	0.85*
*P* Value	0.003*	<0.001*	

SD = standard deviation, *Student *t* test

### Intraocular pressure and success rate

**Fig. 2:**
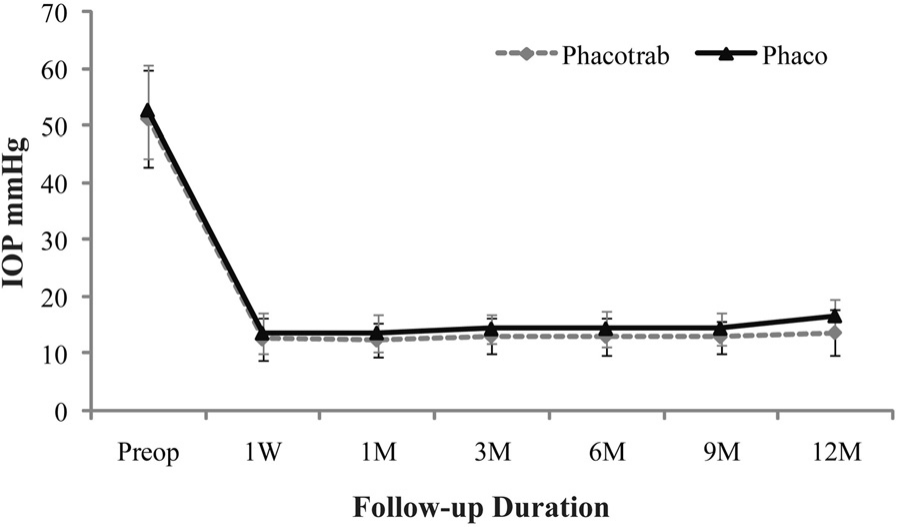
Mean IOP profiles of phacotrab and phaco groups. W: week; M: month; Preop: before surgery

The mean IOP in phacotrab group and phaco group at the time of acute PAC attack were 51.38 ± 8.52 mmHg and 52.60 ± 8.15 mmHg respectively. The mean postoperative IOP levels at all follow up time points were lower than the mean preoperative IOP in each group (*P* <0.001) (Fig. 2). There was statistically significant difference in mean IOP between the two groups only at 12 months postoperatively (*P* = 0.006). The mean postoperative IOP levels at all follow up time points were shown in Table 3.

**Table 3:** Pre- and Post-operative IOP of the two treatment groups

IOP (mmHg)	Phacotrab group	Phaco group	*P* Value
Mean preoperative ± SD	51.38 ± 8.52	52.60 ± 8.15	0.61*
Mean postoperative ± SD (1 week)	12.58 ± 3.74	13.60 ± 3.63	0.34*
1 month	12.38 ± 3.02	13.64 ± 3.21	0.16*
3 months	13.08 ± 3.15	14.36 ± 2.61	0.13*
6 months	13.04 ± 3.18	14.40 ± 3.01	0.13*
9 months	12.96 ± 2.88	14.44 ± 2.83	0.08*
12 months	13.71 ± 3.99	16.64 ± 3.08	0.006*
*P* Value	<0.001*	<0.001*	

SD = standard deviation, *Student *t* test

Starting from the 6 months, 2 eyes required one kind of IOP-lowering drop, and 2 eyes required two to control IOP (≤21 mmHg) in phacotrab group. In this group the success rate was 83.33 % (20 eyes) (without medications) and 100 % (with medications). In phaco group 2 eyes required one kind of IOP-lowering drop, and 5 eyes required two to control IOP. In this group the success rate was 72 % (18 eyes) (without medications) and 100 % (with medications) respectively. 0.5 % timolol was used to control IOP, and then brinzolamide was added when it was not enough to achieve the target IOP.

### Anterior chamber depth and complications

The post-operative mean ACD of the two treatment groups increased (Table 4). No statistically significant differences in the mean ACD were found between the two groups at 6 months. In phacotrab group, bleb needling procedure with 5-fluorouracil (5 mg) was performed in 2 eyes at 3 months. All 24 eyes had diffuse blebs or elevated blebs with microcystic changes in the conjunctiva between 3 months to 12 months. There were no serious intra- or post-operative complications in the two treatment groups.

**Table 4:** Pre- and Post-operative ACD of the two treatment groups

ACD (mm)	Phacotrab group	Phaco group	*P* value
Mean preoperative ± SD	1.60 ± 0.23 (1.08-1.87)	1.59 ± 0.27 (1.16-1.96)	0.93*
Mean postoperative ± SD	2.72 ± 0.30 (2.31-3.22)	2.86 ± 0.28 (2.47-3.35)	0.11*
*P* Value	<0.001*	<0.001*	

SD = standard deviation, *Student *t* test

## Discussion

The timely management of acute PAC is important for reducing the risk of irreversible damage to the optic nerve head and preventing recurrent attacks and chronic angle closure glaucoma (CACG) progression [13]. The retinal fiber layer thickness may decrease significantly within 16 weeks after the attack [14]. Delay in presentation and the time needed to terminate the attack have been found to have a detrimental effect on the final outcome [13]. Conventional options involve the use of medical treatment, paracentesis and laser peripheral iridotomy. In hospital clinics a few patients may be refractory to these treatments and the attack may remain unbroken. Operative options should be considered to lower IOP as soon as possible, but the timing of operations in an acute setting is controversial.

Recently, cyclodiode laser has been described as a safe and effective alternative in the management of medically uncontrolled acute PAC, and the authors demonstrate a good result in five patients only [15]. Most ophthalmologists would consider that lensectomy or trabeculectomy is suboptimal in such situation because of greater risk of operative complications due to the small dimensions of the chamber and the tendency for choroidaleffusion. The complications of lensectomy are: corneal edema, posterior capsular rupture, bleeding, fibrinous inflammatory reaction, and posterior capsular opacification [16]. The complications of trabeculectomy are: shallow anterior chamber, transient IOP elevation, hyphaema, and aqueous misdirection [17]. In medically unresponsive cases of acute PAC, higher risk of surgical failure and complications make trabeculectomy not a preferred choice [17]. In recent times, technological advances in phacoemulsification and small-incision trabeculectomy (SIT) make this option much more viable.

In cases of acute PAC or acute angle-closure glaucoma, phacoemulsification alone has been shown to achieve good IOP control [18–20]. But IOP-spikes may appear in the early postoperative period and pose a potential threat [21]. Phacotrabeculectomy plus intraocular lens implantation has been shown superior than trabeculectomy which is also superior than phacoemulsification in decreasing IOP for primary angle closure-glaucoma (PACG) [22]. Phacotrabeculectomy is more effective than phacoemulsification alone in controlling IOP in medically uncontrolled CACG eyes with coexisting cataract [23]. In eyes with synechial angle closure and cataract, the preferred option is to perform phacotrabeculectomy [24]. With the progress of surgical technique, able to skillfully handle intraoperative and postoperative complications, more and more doctors tend to solve the two problems in combination.

The new procedures and devices aim to lower IOP with a higher safety profile than filtering surgery (trabeculectomy/drainage tubes) are collectively termed “minimally invasive glaucoma surgery (MIGS)” [25]. But these technologies are mainly for open angle glaucoma, surgery for “closed angle” is still dominated by trabeculectomy for Asian eyes [26]. The aim of SIT is to pursue least tissue injury, less complications and better filtering effect. SIT has been introduced in the form of small incision with 3 mm fornix-based conjunctival flap, 1–2 mm short scleral tunnel instead of scleral flap, suture or no suture for incision, reducing operation area and tissue injury [27, 28]. The surgical technique is generally efficacious and relatively safe comparing to the standard trabeculectomy. One study including 41 eyes with medically uncontrolled glaucoma adopted the surgical technique. The glaucoma type included chronic simple glaucoma, chronic narrow-angle glaucoma, pseudoexfoliation glaucoma and pigmentary glaucoma. Most of these patients had IOP at or below the target IOP after mean follow-up of 25 months [29]. Another revised procedure of SIT avoids cutting Tenon’s capsule [30]. The use of a small 2.5 mm limbal incision obviates subconjunctival fibrosis, and it is safer with higher success rate than conventional trabeculectomy [31].

One important difference between the above SIT studies is the glaucoma type, open angle vs. closed angle over 180° in ours. The patients enrolled in our study suffered with both refractory acute PAC and coexisting cataract. We compared small-incision phacotrabeculectomy with phacoemulsification in treating the two problems. Based on the patients’ preoperative status and eye characteristics, we also revised the procedure in order to achieve the best outcome, including the width of the peritomy, flap size, suture method and etc. The BCVA of most patients was improved in phacotrab group (75 %) and phaco group (80 %). The surgical success rate was 83.33 % in phacotrab group and 72 % in phaco group respectively. The difference in mean IOP at 12 months between the two groups appeared marginal. A longer follow-up would be useful to confirm whether small-incision phacotrab is more effective in IOP control.

Merits of small-incision phacotrabeculectomy for refractive acute PAC with cataract include: less postoperative inflammatory reaction as phaco, better IOP control in the long term, less possibility of IOP lowing medication and progression to glaucoma. In addition, combined phacotrabeculectomy may help elderly patients with less psychological and financial burden. Any operative option should base on the specific condition of ocular diseases and the premise of no violation of evidence-based medicine, taking the most advantageous way for patients. In patients with medically uncontrolled glaucoma and cataract, the options are to perform trabeculectomy first then phacoemulsification, phacoemulsification first and then trabeculectomy, or phacotrabeculectomy [32]. The surgical indications of combined phacotrabeculectomy should be reserved for any one of the following conditions: refractory to drug or laser treatment with high IOP, attack history or moderate to severe optic nerve damage, tendency to malignant glaucoma, requirement of vision improvement, no chance to have 2 separate surgeries due to ocular or systemic conditions, and poor adherence or inconvenience of follow-up, etc. Small-incision phacotrabeculectomy may offer clinical and technical advantages over the standard combined operations where conventional treatment fails.

This study may not have sufficient follow-up duration and sample size to look at other parameters, such as additional IOP lowing medication and glaucomatous progression. Multicenter randomized controlled clinical trials are required to confirm these observations.

## Conclusions

Small-incision phacotrabeculectomy may be useful with a lower risk of surgical complications as the primary surgical intervention for the treatment of patients with refractory acute PAC and coexisting cataract.
